# Prevalence of K13-propeller polymorphisms in *Plasmodium falciparum* from China-Myanmar border in 2007–2012

**DOI:** 10.1186/s12936-015-0672-9

**Published:** 2015-04-18

**Authors:** Zenglei Wang, Sony Shrestha, Xiaolian Li, Jun Miao, Lili Yuan, Mynthia Cabrera, Caitlin Grube, Zhaoqing Yang, Liwang Cui

**Affiliations:** Department of Entomology, The Pennsylvania State University, University Park, PA 16802 USA; Department of Pathogen Biology and Immunology, Kunming Medical University, Kunming, Yunnan Province 650500 China

**Keywords:** Malaria, Plasmodium falciparum, artemisinin resistance, *K13* gene, F3D7_1343700

## Abstract

**Background:**

The recent emergence and spread of artemisinin resistance in the Greater Mekong Subregion poses a great threat to malaria control and elimination. A K13-propeller gene (*K13*), *PF3D7_1343700*, has been associated lately with artemisinin resistance both *in vitro* and *in vivo*. This study aimed to investigate the *K13* polymorphisms in *Plasmodium falciparum* parasites from the China-Myanmar border area where artemisinin use has the longest history.

**Methods:**

A total of 180 archived *P. falciparum* isolates containing 191 parasite clones, mainly collected in 2007–2012 from the China-Myanmar area, were used to obtain the full-length *K13* gene sequences.

**Results:**

Seventeen point mutations were identified in 46.1% (88/191) parasite clones, of which seven were new. The F446I mutation predominated in 27.2% of the parasite clones. The C580Y mutation that is correlated with artemisinin resistance was detected at a low frequency of 1.6%. Collectively, 43.1% of the parasite clones contained point mutations in the kelch domain of the *K13* gene. Moreover, there was a trend of increase in the frequency of parasites carrying kelch domain mutations through the years of sample collection. In addition, a microsatellite variation in the N-terminus of the *K13* protein was found to have reached a high frequency (69.1%).

**Conclusions:**

This study documented the presence of mutations in the *K13* gene in parasite populations from the China-Myanmar border. Mutations present in the kelch domain have become prevalent (>40%). A predominant mutation F446I and a prevalent microsatellite variation in the N-terminus were identified, but their importance in artemisinin resistance remains to be elucidated.

## Background

Malaria has been scourging human beings for millennia, and remains responsible for over 430,000 child deaths in Africa every year [1]. The world has made remarkable strides in battling against this ancient enemy during the past decade, reducing by an impressive 47% in mortality rate globally between 2000 and 2013 [1]. However, parasite resistance to anti-malarials remains an ever-present obstacle to eliminate malaria. Chloroquine and sulphadoxine-pyrimethamine have failed as crucial medicines in the treatment of the deadly malaria parasite *Plasmodium falciparum* due to the emergence and rapid spread of drug resistance. More worryingly, resistance to artemisinin (ART) family drugs has been detected and is spreading in Southeast Asia [2-4], posing a major threat to the implementation of artemisinin-based combination therapy (ACT) as a defensive line against *P. falciparum*.

ART resistance is manifested clinically as delayed parasite clearance half-life (>5 hours) *in vivo* [4,5]. An *in vitro* ring-stage survival assay (RSA_0-3h_), which measures the percentage of early ring-stage parasites (0–3 hrs post-invasion of red blood cells (RBCs)) that survive exposure to a pharmacologically relevant concentration of dihydroartemisinin, has been developed to reflect this ART resistance phenotype [6]. Recent work has associated ART resistance with mutations in the propeller domain of a kelch gene on chromosome 13 (*PF3D7_1343700*, *K13* gene) [5]. The *K13* mutation M476I was initially identified in a Tanzanian *P. falciparum* strain that had undergone *in vitro* ART selection for five years. Research on parasite isolates from Cambodia, where ART resistance was first observed, identified *K13* mutations Y493H, R539T and C580Y to be associated with delayed clearance [5]. These mutations were confirmed to contribute to *in vitro* ART resistance through genetic manipulations of the *K13* gene [7,8]. A large, multicentre, clinical study further indicates that ART resistance is spreading in the Greater Mekong Subregion (GMS), where single-point mutations in the propeller domain of *K13* after the position 440 are collectively associated with ART resistance [4]. Surveys conducted in different regions showed that *K13* mutations associated with ART resistance were restricted to certain areas of the GMS, including Cambodia, Thailand, Myanmar, and Vietnam. The C580Y mutation is the predominant one approaching fixation in Western Cambodia [5,9-11]. These mutations have not been detected in Bangladesh and Laos [4,10,12]. Surveys of African parasite populations, while having found a diverse array of mutations within the *K13* gene, did not detect those mutations associated with ART resistance [13-17].

ART family drugs have been used in China’s Yunnan Province since the late 1970s [18]. In recent years, clinical efficacy studies conducted in this region showed that artemisinin drugs for treating falciparum malaria remain highly effective [19,20]. However, the proportion of day 3 parasite-positive cases in one study reached 18.5% [20], well above the 10% threshold set by the World Health Organization as a proxy indicator of suspected ART resistance [21], suggesting possible emergence of ART resistance in this area. In this study, the polymorphisms of *K13* genes in parasite populations along the China-Myanmar border were investigated, and the presence of *K13* mutations that are associated with clinical ART resistance in this region was demonstrated using longitudinally archived parasite samples. More importantly, parasite strains carrying wild-type *K13* alleles have been declining through the six years of sample collection.

## Methods

### Collection of parasite clinical isolates, DNA extraction and genotyping

*Plasmodium falciparum* clinical isolates were collected during the period 2004–2012 in malaria clinics located along the China-Myanmar border, cultured and archived [22]. A total of 180 samples were analysed including two collected in 2004, 25 in 2007, 47 in 2008, 78 in 2009, 11 in 2010, five in 2011, and 12 in 2012. Genomic DNA was extracted using the Wizard® Genomic DNA Purification Kit (Promega, WI, USA). Parasite samples were genotyped at *msp1*, *msp2* and *glurp* using previously described methods [23-25] to distinguish single from mixed-strain infections [26].

### *K13* propeller gene amplification and sequencing analysis

The full-length *K13* gene was amplified by high-fidelity PCR using Advantage HD DNA Polymerase Mix (Clontech, Mountain View, CA, USA) and primers KP13-F (5′-TATAACAAGGCGTAAATATTCGTG-3′) and KP13-R (5′-TGTGCATGAAAATAAATATTAAAGAAG-3′). PCR reactions were performed in 37.5 μl with 0.5 μM of DNA template, 0.2 μM of each primer, 3.75 μl of 10 × PCR buffer, 3.75 units of DNA polymerase mix, and 0.2 mM of dNTP mix. Reaction conditions consisted of an initial denaturation at 95°C for 5 min followed by 35 cycles of 95°C for 30 sec, 55°C for 30 sec and 68°C for 3 min, and a final extension step for 7 min at 68°C. PCR products were sequenced in both directions using sequencing primers KP13-65 F (5′-GGGAATCTGGTGGTAACAGC-3′), KP13-640R (5′-CACTAGCATCACTTAATTCCGTT-3′), KP13-517 F (5′-GATGCAGCAAATCTTATAAATGATG-3′), KP13-759 F (5′-GGAAAGAGTACGATTGTACAAAG-3′), KP13-1363R (5′-CTACACCATCAAATCCACCTATA-3′), KP13-1595 F (5′-GTGGTGTTACGTCAAATGGTAG-3′), and KP13-R. Sequences were assembled by DNASTAR (WI, USA) with manual editing. Alignment of DNA sequences were performed using MEGA 6.0 [27] with the *K13* sequence of the 3D7 clone (*PF3D7_*1343700) retrieved from PlasmoDB as the reference. To superimpose the mutations in the *K13* protein, the 3D structure of the *K13* protein was predicted by Phre2 online protein structure prediction tool [28]. The chosen template retained 100% confidence based on homology assessment and model prediction quality.

### Statistical analysis

Fisher’s exact test was done to assess difference in the frequency of mutations between years using GraphPad Prism 5 (GraphPad Software, Inc. La Jolla, CA, USA).

## Results

The full-length *K13*-propeller genes were sequenced from a total of 180 clinical isolates collected from malaria patients along the China-Myanmar border. Of these isolates, 178 were collected during 2007–2012, while two were collected in 2004. Genotyping of these parasites at three polymorphic genes confirmed that 169 were monoclonal infections, whereas 11 were mixed infections at one of the three loci [29]. Since each of the mixed infections contained only two allelic types, they were considered as having two parasite strains in each sample. Based on their clear sequencing chromatograms, these mixed infections were also included in the analysis. Accordingly, the frequencies of mutations were estimated by using 191 parasite clones in the study population.

**Figure 1:**
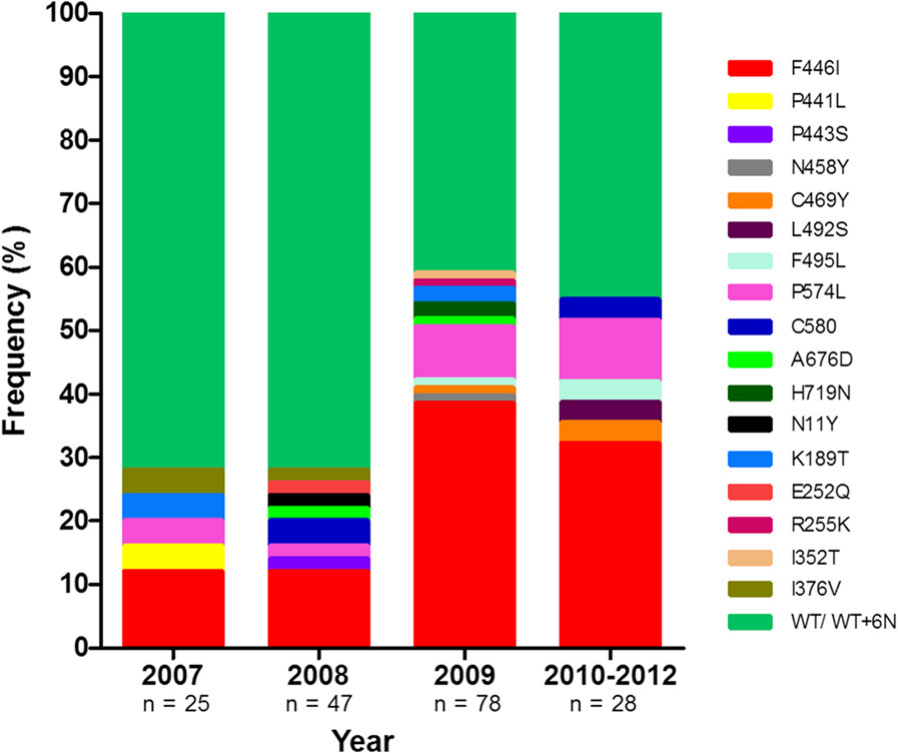
Prevalence of *K13* mutations in parasites collected from the China-Myanmar border area in 2007–2012.

Sequencing of the *K13* genes in the 191 clones revealed that 88 (46.1%) contained single nucleotide polymorphisms (SNPs) at 17 locations. All 17 SNPs were non-synonymous; no synonymous mutation was detected in these isolates. In addition, variations in the number of a microsatellite repeat (ATA) corresponding to amino acid positions 137–142 of *K13* in 3D7 parasite were also observed. This microsatellite sequence encodes the Asn (N) residue and is present in 30.4, 69.1 and 0.5% of the 191 clones as six (wild type), eight and nine Ns, respectively. For the point mutations, ten (K189T, E252Q, R255K, P441L, F446I, N458Y, P574L, C580Y, A676D and H719N) were described previously [4,5,30,31] and seven (N11Y, I352T, I376V, P443S, C469Y, L492S and F495L) were new to parasites in this region (Table 1, Figure 1). All of these mutations occurred singly, except that a double-mutation P574L and F446I was detected in one parasite clone. One major distinction of this parasite population is the predominant status of the F446I substitution, reaching a frequency of 27.2%. Coincidently, this point mutation was only observed in parasites with the 8 N repeats at the microsatellite locus. The P574L mutation was the second most prevalent (6.7%). The rest of mutations were all rare and occurred in one to three clones (0.5–1.6%). It is noteworthy that the C580Y mutation, which was correlated with ART resistance and prevailed in other areas of Southeast Asia, was present in only three clones (1.6%). None of the I543T, R539T and Y493H mutations associated with delayed parasite clearance in the Cambodian isolates, or the M476I substitution selected *in vitro* in a Tanzanian strain, was observed in this parasite population.

**Table 1:** **Amino acid substitutions in the** ***K13*** **gene in parasites from the China**-**Myanmar border** (**n** = **191**)

**Mutation**	**No (%)**
N11Y*	1 (0.5)
K189**T**	3 (1.6)
E252**Q**	1 (0.5)
R255**K**	1 (0.5)
I352T*	1 (0.5)
I376V*	2 (1.0)
P441**L**	1 (0.5)
P443S*	1 (0.5)
F446**I**	52 (27.2)
N458**Y**	1 (0.5)
C469Y*	2 (1.0)
L492S*	1 (0.5)
F495L*	2 (1.0)
P574**L**	12 (6.3)
C580**Y**	3 (1.6)
A676**D**	2 (1.0)
H719**N**	2 (1.0)
In kelch domain	79 (41.3)
Total	88 (46.1)

*indicates unique mutations identified in this parasite population. Letters in bold indicate mutated amino acids.

**Figure 2:**
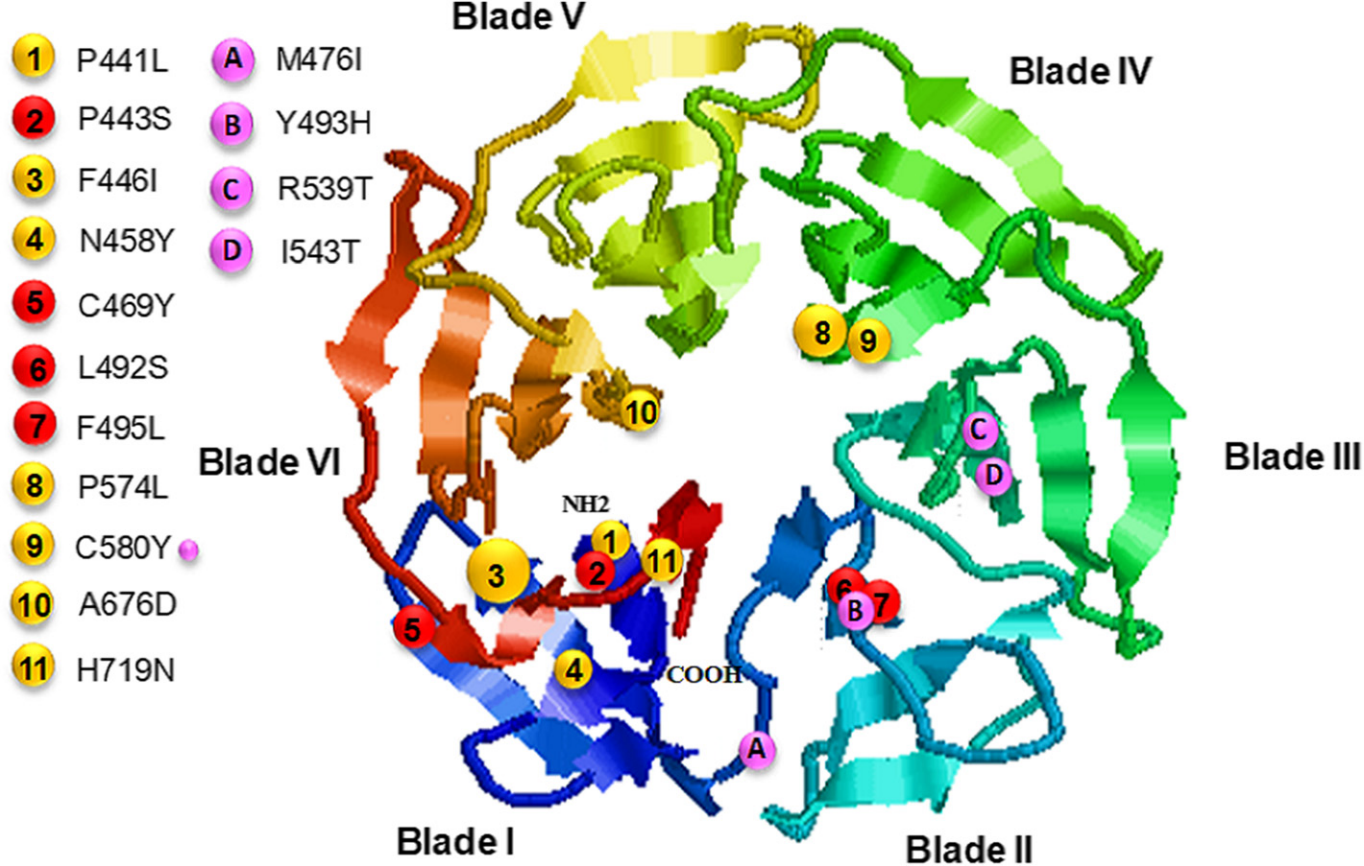
Distribution of the mutations in the predicted 3D model of the *K13* propeller domain. The predicted structure of the propeller domain forms six propeller blades that contain predominantly strands. The locations of the various mutations are indicated by spheres, where red colour represents new mutations, orange for previous reported mutations, and pink for mutations correlated with ART resistance reported by Ariey *et al.* The relative frequencies of the mutations are reflected by the size of the spheres.

**Figure 3:**
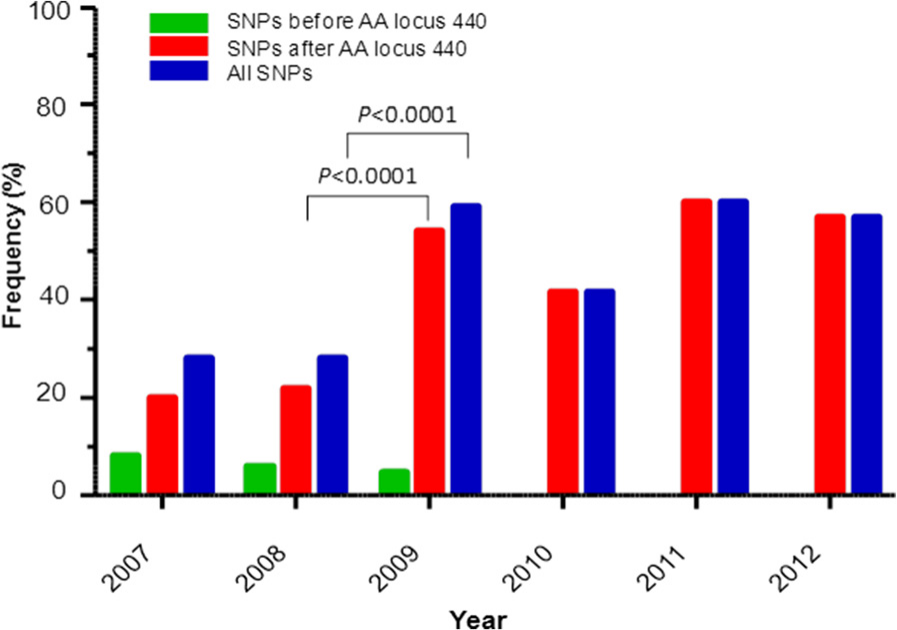
Frequencies of *K13* mutations in 2007 to 2012. Significant increase in frequencies of mutations in the kelch domain in 2009 was noted.

The *K13* protein contains a piece of *Plasmodium*-specific sequence and a BTB/POZ domain in the N-terminus, and a 6-blade propeller domain in the C-terminus (codons 441–725) [5]. Of the 17 mutations identified in this parasite population, six were distributed in the N-terminus in nine clones, whereas the remaining 11 occurred within the kelch propeller domain in 41.3% parasite clones. Superimposing the mutations to the predicted kelch domain structure showed that five (P441L, P443S, F446I, N458Y, C469Y) were clustered in blade I, two (L492S and F495L) in blade II, two (P574L and C580Y) in blade IV, and two (A676D and H719N) in blade VI (Figure 2). A recent multicentre clinical investigation showed that various mutations occurring in the kelch domain after position 440 are collectively associated with an increase in parasite clearance half-life [4]. When the samples were stratified by the year of collection, the frequencies of all mutations in the kelch domain, as well as all mutations in *K13* gene, increased during the time 2007–2012 (Figure 3). In particular, a significant increase was observed in the frequencies of mutations in the kelch domain between 2008 and 2009 (*P* <0.0001). However, the frequencies of mutations in the N-terminus of K13 protein decreased over the years of collection (Figure 3).

## Discussion

The involvement of *K13* mutations in ART resistance in Cambodia encouraged molecular surveillance of *K13* genes in many malaria-endemic regions. *K13* mutations associated with ART resistance were mainly detected in areas of the GMS [5,9-11], with C580Y being the predominant. In contrast, surveys in Africa identified numerous mutations in the *K13* gene, but most occurred at low frequencies. Moreover, mutations associated with ART resistance were not observed [13-16]. In this study, sequencing of *K13* gene from 180 longitudinally collected parasite samples (containing 191 parasite clones) from the China-Myanmar border area identified 17 mutations (ten previously described and seven new). Compared with other surveys in Myanmar or the China-Myanmar border, parasites from this study shared three mutations (F446I, P574L and A676D) with those reported by Feng *et al.* [30], three (P441L, N458Y and C580Y) with those reported by Nyunt *et al.* [9] and four (F446I, P574L, C580Yand A676D) with those reported by Tun *et al.* [31]. Of particular interest is the identification of the F446I as the predominant mutation in the study samples, which has reached a frequency of 27.1%, coincident with results from other study (19.2%) in this region [30]. Although other mutations were at low frequencies, they collectively gave a total of 41.3% parasites carrying mutations in the kelch domain. Among them, the predominant C580Y mutation in other regions of Southeast Asia [4,5,10] was detected in 1.6% of these samples. This mutation was found to be significantly associated with prolonged parasite clearance half-life in parasites from Cambodia [5] and was confirmed to confer ART resistance by genetic manipulations [7,8]. Most of the mutations in the kelch domain, including the most prevalent F446**I** mutation, remain to be genetically characterized to determine whether they confer ART resistance.

Another intriguing phenomenon discovered in this study is the highly prevalent microsatellite variations in the N-terminus of the *K13* gene. Compared to the wild-type parasite, which has six N residues, 69.1% parasites harboured eight N residues. The eight N variation was only reported in Senegalese isolates with a frequency of 6.3% [16]. More interestingly, the predominant mutation F446I was observed only in parasites with eight N repeats. In an earlier study, microsatellite variations in the *nhe1* gene were associated with altered sensitivities to quinine in parasite population from the same area [26]. Therefore, it would be interesting to find out whether the *K13* microsatellite variations affect parasites’ sensitivities to ART drugs.

ARTs have been used in the China-Myanmar border area for over three decades, mostly as monotherapy prior to 2005. The ACT drug deployed here is the dihydroartemisinin/piperaquine combination, compared to artesunate/mefloquine in most other areas of the GMS. One can hypothesize that it is likely that the distinct *K13* mutations observed in parasites from this study site might have emerged separately as a result of selection from a different ART regimen. In addition, given the predominant status of the C580**Y** mutation in other parts of the GMS, it remains an open question as to whether the presence of this mutation in these samples was due to independent emergence or had spread from other areas. Furthermore, clinical efficacy studies also detected day-3 parasite positivity rate to >10% after artesunate treatment of falciparum malaria in this area [32]. Taken together, increased surveillance and population studies are needed to determine further spread of ART resistance in the GMS.

## Conclusions

Seventeen point mutations were identified in the *K13* gene from 180 archived parasite samples collected from the China-Myanmar border area. Analysis of the 191 parasite strains identified 43.1% carrying point mutations in the kelch domain, of which the F446I mutation reached a predominance of 27.1%. Given the presence of *K13* mutations conferring ART resistance and detection of day-3 parasite positivity after ART drug treatment, increased surveillance of *P. falciparum* for anti-malarial drug resistance is highly demanded.
